# Thermal Operational Stability in Organic Thin Film Transistors: The Critical Role of Interface Composition and Deposition Conditions

**DOI:** 10.1002/smtd.202502171

**Published:** 2026-02-28

**Authors:** Forest St‐Denis‐Weintrager, Sofia Gallardo‐Pascual, Nicolas Ledos, Raluchukwu B. Ewenike, Audithya Nyayachavadi, Halynne R. Lamontagne, Benoît H. Lessard

**Affiliations:** ^1^ Department of Chemical and Biological Engineering University of Ottawa Ottawa Ontario Canada; ^2^ Department of Chemistry and Biomolecular Sciences University of Ottawa Ottawa Ontario Canada; ^3^ School of Electrical Engineering and Computer Science University of Ottawa Ottawa Ontario Canada

**Keywords:** interfacial engineering, organic thin‐film transistors (OTFTs), phthalocyanine, self assembled monolayer, thermal stability

## Abstract

The widespread adoption of organic thin‐film transistors (OTFTs) in flexible, wearable, and biodegradable electronics depends on achieving optimal electrical performance while ensuring reliable performance under typical environmental stressors such as humidity, oxygen, and heat. Among these, thermal stability remains underexplored, despite being critical for devices exposed to sterilization, body heat, or fluctuating environmental conditions. Here, we demonstrate that the thermal response of fluorinated silicon phthalocyanine (F_10_‐SiPc) OTFTs can be rationally tuned by interfacial chemistry and deposition temperature, providing a scalable route to environmental robustness. By comparing octyltrichlorosilane (OTS), hexamethyldisilazane (HMDS), and plasma‐treated SiO_2_ dielectrics with F_10_‐SiPc films deposited at either 25°C or 100°C, we identify two distinct transport regimes. OTS promotes highly crystalline films with excellent room‐temperature mobility but exhibits non‐recoverable reduced performance at elevated operating temperatures, accompanied by increased post‐heating hysteresis, suggestive of dipole‐induced interfacial charge trapping. In contrast, HMDS and plasma yield more disordered morphologies that reorganize reversibly under heat, leading to mobility enhancements of 100%–800% via thermally activated hopping. Raising the deposition temperature lowers the initial mobility but reshapes the trajectory of thermal response. Together, these results establish interlayer selection and deposition temperature as complementary design parameters to deliberately program thermal stability profiles in OTFTs. This framework connects processing to operational reliability, advancing the design of organic electronics capable of surviving real‐world thermal and environmental demands without new materials or complex encapsulation.

## Graphical abstract

Operational thermal stability in organic thin‐film transistors is engineered through interfacial chemistry and deposition temperature. By pairing F10‐SiPc with different surface treatments and tuning substrate temperature during deposition, distinct transport regimes emerge, ranging from crystalline, trap‐prone behavior to reversible, thermally activated mobility gains. This approach provides a scalable strategy to program environmental robustness without new materials or complex encapsulation.

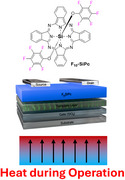

## 1 Introduction

Organic semiconductors are promising for scalable, high‐volume flexible electronics [1], low‐cost sensors [2, 3, 4], and biodegradable devices [5, 6]. In these applications, device performance must withstand environmental stressors such as air exposure and elevated temperatures [7]. Over the past two decades, OTFT performance has advanced substantially, with recent cases surpassing benchmark amorphous silicon devices [8]. However, their susceptibility to degradation in ambient conditions remains a major barrier to commercialization [9]. Several studies on polymers and small molecules have examined the influence of oxygen and humidity on device performance, yet most recent research focuses primarily on intrinsic material parameters, often overlooking the thermal operational stability of the devices. Generally, when the operational stability of organic semiconductors integrated into OTFTs has been considered, the effect of operating temperature has received less attention compared to air stability [10, 11].

Charge transport in organic semiconductors has been widely studied through theoretical and experimental approaches, often under sub‐room‐temperature conditions to isolate specific transport mechanisms [12, 13, 14, 15]. While these studies have deepened the fundamental understanding of charge transport, it is equally important to evaluate general device performance under conditions relevant to practical operation. Three main strategies have been reported to improve the thermal stability of organic electronic devices. The first involves the use of encapsulation layers to protect devices from high‐temperature‐induced degradation. For example, Kuribara et al. developed thermally stable DNTT‐based transistors for medical applications, employing a parylene–gold–parylene encapsulation that preserved device performance during harsh sterilization processes above 100°C [16]. The second strategy focuses on designing semiconducting and conducting materials with intrinsic resistance to high temperatures [17, 18, 19, 20]. Cho et al. demonstrated thermally stable OFETs using the amorphous polymer PCDTBT, which maintained its electronic properties when heated above its glass‐transition temperature, in contrast to typical semicrystalline conjugated polymers [17]. Similarly, a high‐mobility polymer FET based on PCDTPT retained its electronic properties after exposure to 350°C in N_2_ and 200°C in air, a stability attributed to its relatively high HOMO level and rigid fused‐ring backbone [20]. The third strategy leverages molecular additives: Nikolka et al. demonstrated that inserting small‐molecule additives into conjugated polymer films displaces water from nanoscopic voids, dramatically enhancing both operational and environmental stability without sacrificing mobility [21]. While these strategies continue to be optimized, it is notable that the influence of fabrication parameters such as substrate surface chemistry and deposition temperature on thermal resistance has received little attention, despite their well‐established impact on crystal growth and thin‐film electrical properties. Toward advancing our understanding of stability for OTFTs, this study evaluates the key performance metrics of the selected material and examines how these parameters affect device performance under elevated temperatures up to 100°C. Crucial applications, including wearable devices, must be designed to operate consistently when exposed to climate variation and human skin contact, which do not exceed 100°C [22, 23]. Optimizing thermal fabrication conditions offers a practical, scalable route to improve high‐temperature stability without developing new materials, introducing additives, or optimizing complex encapsulation procedures.

**FIGURE 1:**
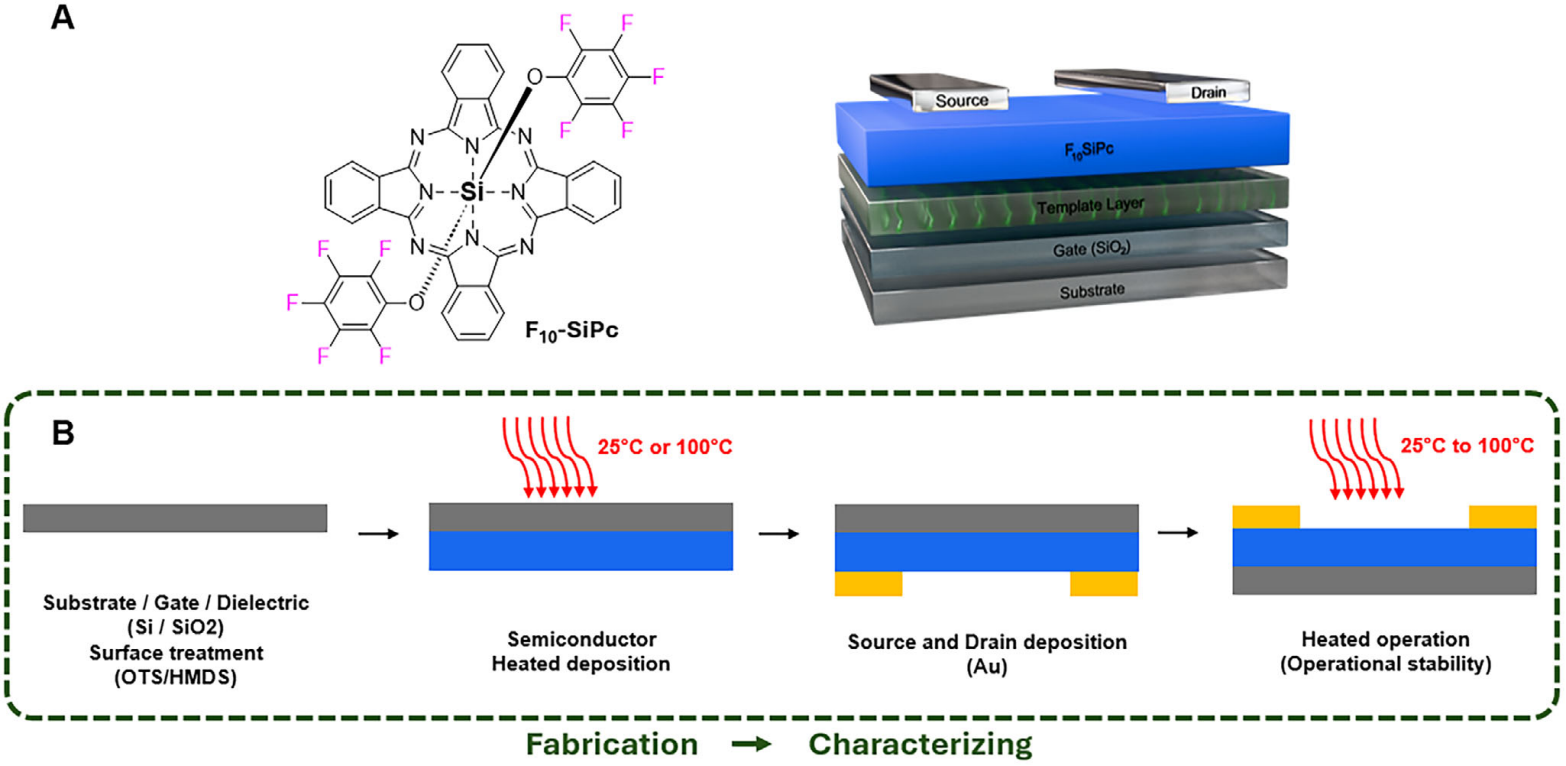
(a) Chemical structure of bis(pentafluorophenoxy) silicon phthalocyanine (F_10_‐SiPc) and a diagram of a bottom gate, top contact organic thin film transistor (OTFT), (b) schematic of the fabrication and operation/stability assessment of OTFTs. The F_10_‐SiPc layer and electrodes are 50 nm in thickness.

Bis(pentafluorophenoxy) silicon phthalocyanine (F_10_‐SiPc, Figure 1) was chosen as a semiconductor for this study because, when deposited on an OTS‐treated SiO_2_ surface, it has achieved high field‐effect mobilities ranging from 0.1 to 0.5 cm^2^ V^−1^ s^−1^ [24, 25]. OTS and HMDS were chosen as surface treatments due to their previously reported ability to enhance the electrical performance of F_10_‐SiPc, attributed to their high hydrophobicity, while plasma‐treated substrates were included to represent a lower surface energy condition [26]. The OTFTs were fabricated by physical vapor deposition (PVD) at two different substrate temperatures, room temperature (RT) and 100°C, to tune the initial film crystallinity. Electrical characterisation of OTFTs operated from 25°C to 100°C revealed that by changing the fabrication parameters, the electrical behavior of the organic semiconductor under exposure to different operating temperatures is significantly affected. In situ Grazing‐Incidence Wide‐Angle X‐Ray Scattering (GIWAXS) measurements were used to correlate the electrical properties to changes in crystalline structure, showing high dependence on the surface treatment. This study demonstrates that the choices in substrate preparation for organic electronic devices influence the final thermal operational stability.

## 2 Results and Discussion

### 2.1 Effect of Deposition Temperature and Surface Treatment

**FIGURE 2:**
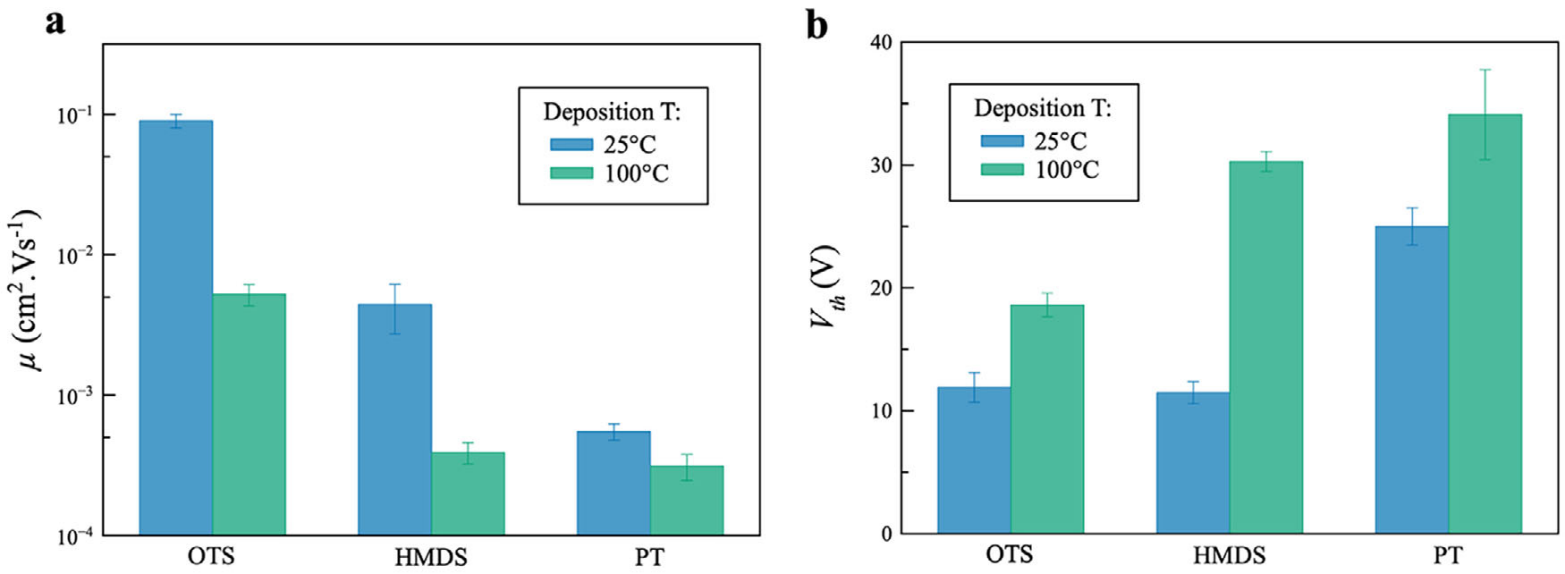
Preliminary electrical performances, including (a) charge carrier mobility (*µ_e_*), and (b) threshold voltage (*V_th_*), for OTFTs operated at room temperature, for devices with F_10_‐SiPc deposited on substrates at 25°C (blue) and 100°C (green).

F_10_‐SiPc‐based BGTC OTFTs were fabricated using silicon substrates by first treating the silicon substrates with a layer of thermally grown silicon dioxide, followed by plasma (PT) and/or the coupling with HMDS, or OTS as a surface treatment. Using PVD, F_10_‐SiPc was then deposited on the different substrates, which were held at 25°C or 100°C during deposition. Silver electrodes with a manganese layer were then deposited by PVD. Using our Gen 1H auto tester, we were able to characterize 20 OTFT devices sequentially on the same semiconducting film at room temperature [27]. Electrical performance characteristics, including average electron mobility (*µ_e_*) and threshold voltage (*V_th_*) for OTFTs with varying template layer and deposition temperature, are characterized at room temperature to obtain a device performance baseline (Table 1, Figure 2).

The crucial role of surface chemistry is highlighted in the significant differences in performance between OTS, HMDS, and PT‐treated devices. For devices fabricated using 25°C or 100°C deposition temperatures, the *µ_e_* of OTS‐treated devices is over an order of magnitude higher than that of PT and HMDS‐treated devices. At both 25°C and 100°C, *V_th_* values for OTS‐ (11.5 V, deposited at 25°C) and HMDS‐treated films (11.9 V, deposited at 25°C) are substantially lower than for PT‐treated films (25 V, deposited at 25°C). The superior *µ_e_* obtained for OTS‐treated devices has been previously reported by our group [28]. Consistent with our previous findings, the *µ_e_* of SiPc thin films deposited by PVD remains positively correlated with the hydrophobicity (*q_w_*) of the interlayer, as *q_w_* (OTS) > *q_w_* (HMDS) > *q_w_* (PT) [29, 30]. Previous studies from our group have characterized the water contact angle on OTS and HMDS‐treated Si/SiO_2_ substrates, finding that OTS achieves a contact angle between 99° and 101° and HMDS achieves a lesser contact angle of 85°–89° [31]. The temperature of the substrate during deposition also has a strong impact on the electrical performance. Thin films deposited at 100°C exhibit *µ_e_* values an order of magnitude lower than at 25°C (Figure 2), which is consistent with the reported effect of deposition temperature on crystal growth, film morphology, and charge transport [32, 33].

#### 2.1.1 Morphology of Initial Films

**FIGURE 3:**
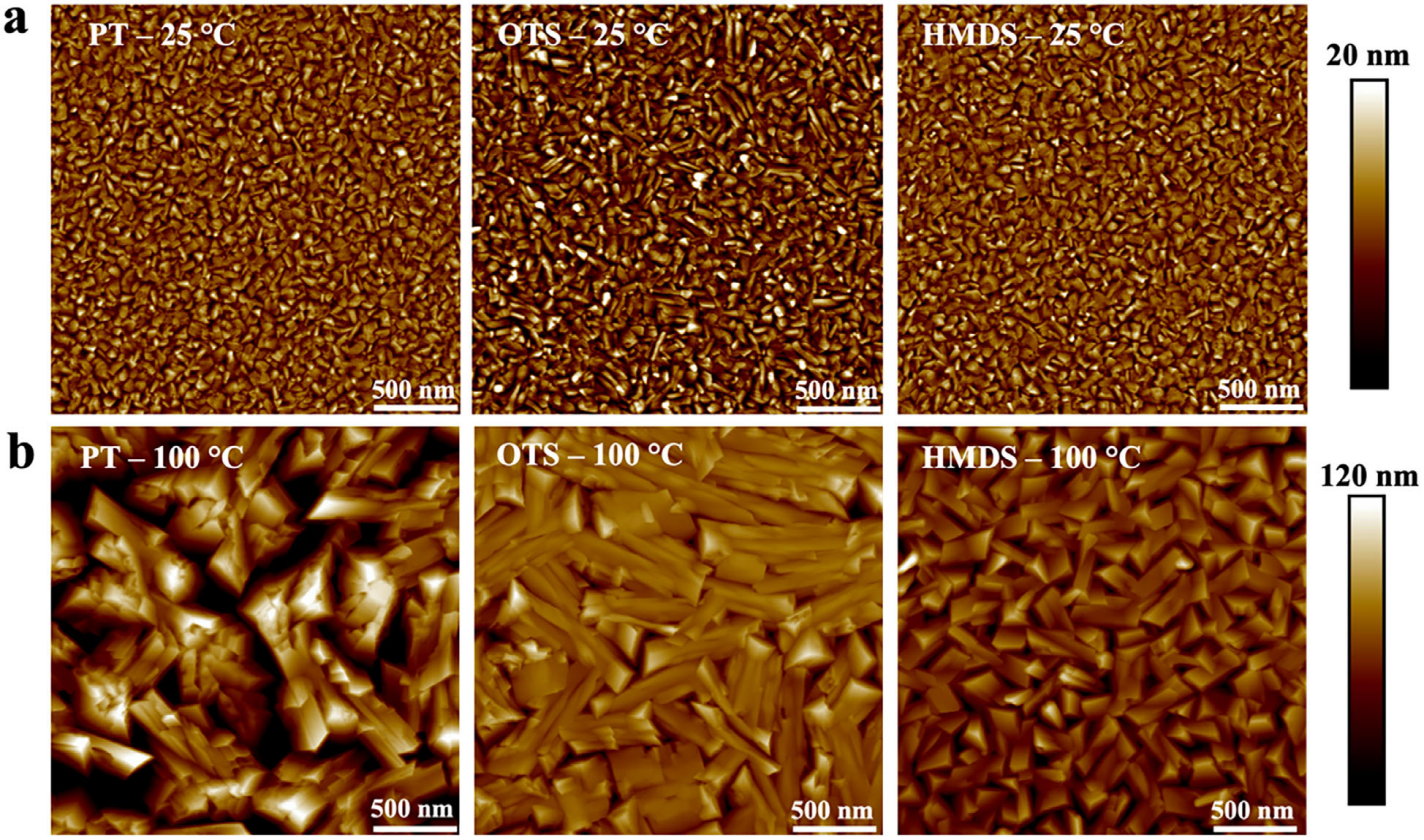
AFM images of F_10_‐SiPc films deposited on plasma, OTS, and HMDS treated substrates (from left to right) at (a) 25°C, and (b) 100°C. All images are 2.5 by 2.5 µm.

Since F_10_‐SiPc was the only semiconductor used throughout this study, differences in device performance must originate from variations in film morphology and interfacial surface layers. To examine this, we used atomic force microscopy (AFM) to analyze the effects of surface treatments and substrate temperature during deposition on the resulting morphology of the F_10_‐SiPc semiconductor layer (Figure 3).

Figure 3 shows that substrate deposition temperature strongly influences F_10_‐SiPc grain size, with 25°C films exhibiting fine grains and 100°C films displaying larger, discrete‐shaped morphologies. This trend is consistent with previous reports on metal phthalocyanines [20, 21, 22, 23]. At elevated temperatures, the higher kinetic energy of molecules promotes migration to lower‐energy sites, creating nucleation sites and yielding polycrystalline films with larger crystallites [32, 34, 35, 36, 37]. The prominent increase in crystallite size and the corresponding enlargement of grain boundaries observed in the thin films fabricated at 100°C could account for the overall decrease in *µ_e_* and increase in *V_th_* measured during electrical characterization. Larger crystallites often lead to fewer grain boundaries per unit area; however, the boundaries that do form can become more irregular and defective, creating energetic barriers and trap sites that hinder charge carrier transport. These traps can reduce effective *µ_e_* by scattering or localizing carriers, while the increased density of localized states near the grain boundaries can shift the *V_th_* to higher values [38]. The RMS roughness values were determined for the films fabricated on substrates at room temperature and 100°C, as summarized in Table 2.

A significant increase in surface roughness was observed for the films fabricated at 100°C, which has been correlated with a reduction in the field‐effect mobility (*µ_e_*).

Surface treatment also influenced morphology: at 25°C, HMDS‐ and plasma‐treated substrates yielded small, randomly oriented isotropic grains, whereas OTS treatment produced regular, rounded anisotropic (ellipsoid) grains. This trend persisted at 100°C, underscoring the role of substrate treatment in film morphology, which we previously correlated with enhanced charge transport when grains align along the favored crystallographic axis (µa) [39].

### 2.2 Effect of Operating Temperature on Electrical Characterization

**FIGURE 4:**
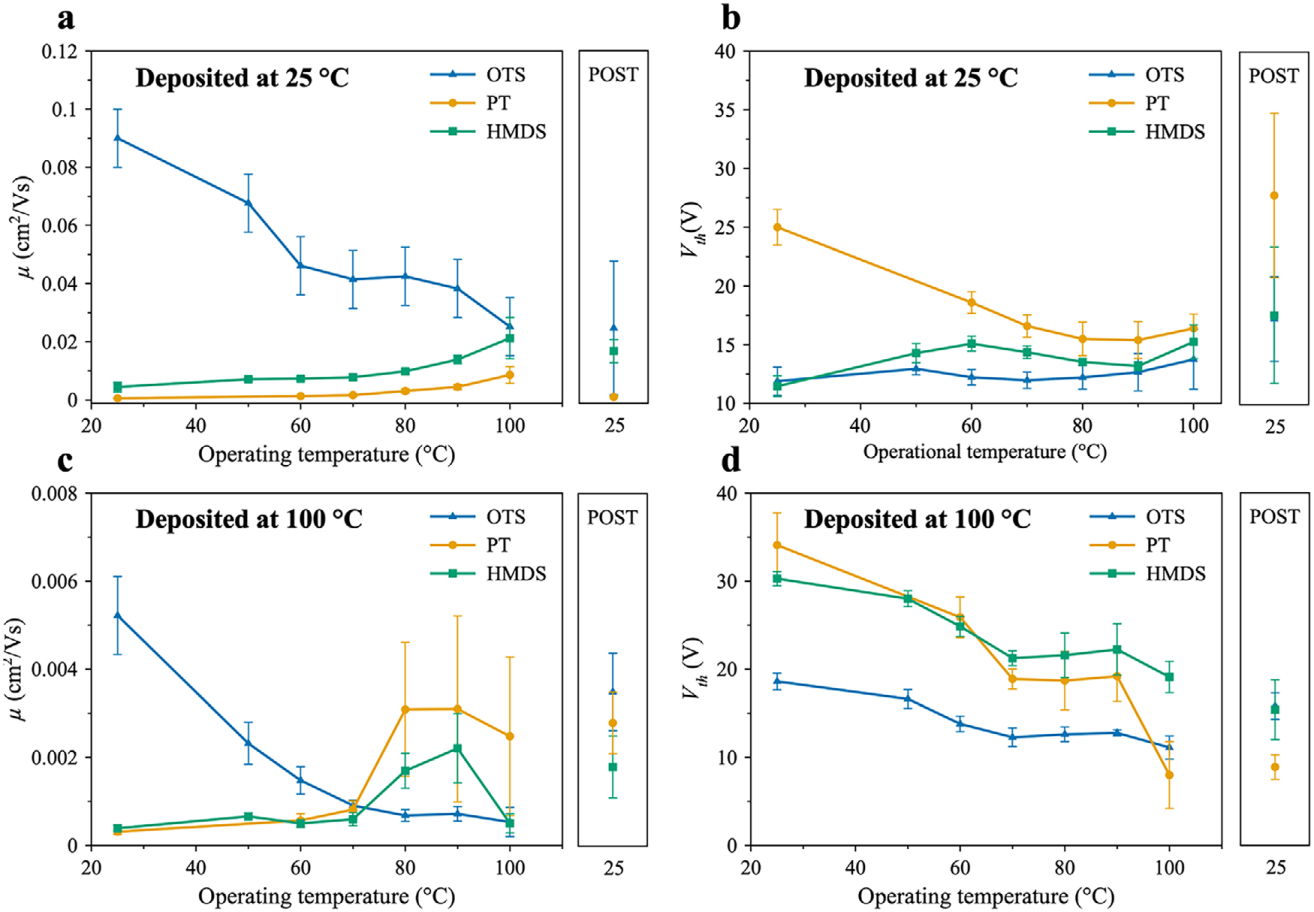
(a,c) Pre‐ and post‐heated testing charge carrier mobility (*µ_e_*) values for F_10_‐SiPc‐based OTFTs deposited on OTS (blue), PT (yellow), and HMDS (green) treated SiO_2_ at substrate temperatures of 25°C and 100°C, respectively. (b,d) Pre‐ and post‐heated testing threshold voltage (*V_th_*) values for the same conditions. Error bars are based on standard deviations, with *N* = 9–12.

To analyze the performance of the F_10_‐SiPc‐based OTFTs at elevated operating temperatures, output and transfer curves were measured in situ as a function of temperature through a programmed ramp soak profile from 25°C to 100°C using an in‐house Gen 1H auto tester [27]. For the characterization of devices operated above 100°C, further studies must be conducted. Electrical performance characteristics, including *µ_e_* and *V_th_* for all 6 sets of OTFTs, are reported in Figure 4. OTFT output and transfer characteristics follow the Shockley transistor model [40] at all temperatures for all the conditions (Figures S2 and S3).

For devices fabricated at both 25°C and 100°C, OTS‐treated devices showed a pronounced decrease in *µ_e_* with increased operating temperature, for example, from 0.085 to 0.024 cm^2^ V^−1^ s^−1^ (deposited at 25°C, Figure 4a). For PT‐ and HMDS‐treated devices, *µ_e_* increased with operating temperature, indicating that electrical performance depends strongly on surface treatment. All devices show a decrease in *V*_th_ with increasing operating temperature, with the drop more pronounced in films fabricated at 100°C than in those deposited at 25°C (Figure 4b,d). This pronounced drop in *V_th_* for the devices with films fabricated at 100°C could be attributed to the larger grain boundaries present in the films. At room temperature, the devices exhibit higher *V_th_* values due to charge traps, as the temperature increases, the thermal activation empties these charge traps more effectively, causing a sharper drop in *V_th_* [44]. Contact effects could also be playing a role in the shifts observed on the *V_th_*. However, the differences between the conditions were attributed to surface treatment and substrate temperature during deposition, since the electrode material and semiconductor are the same for all the conditions.

**FIGURE 5:**
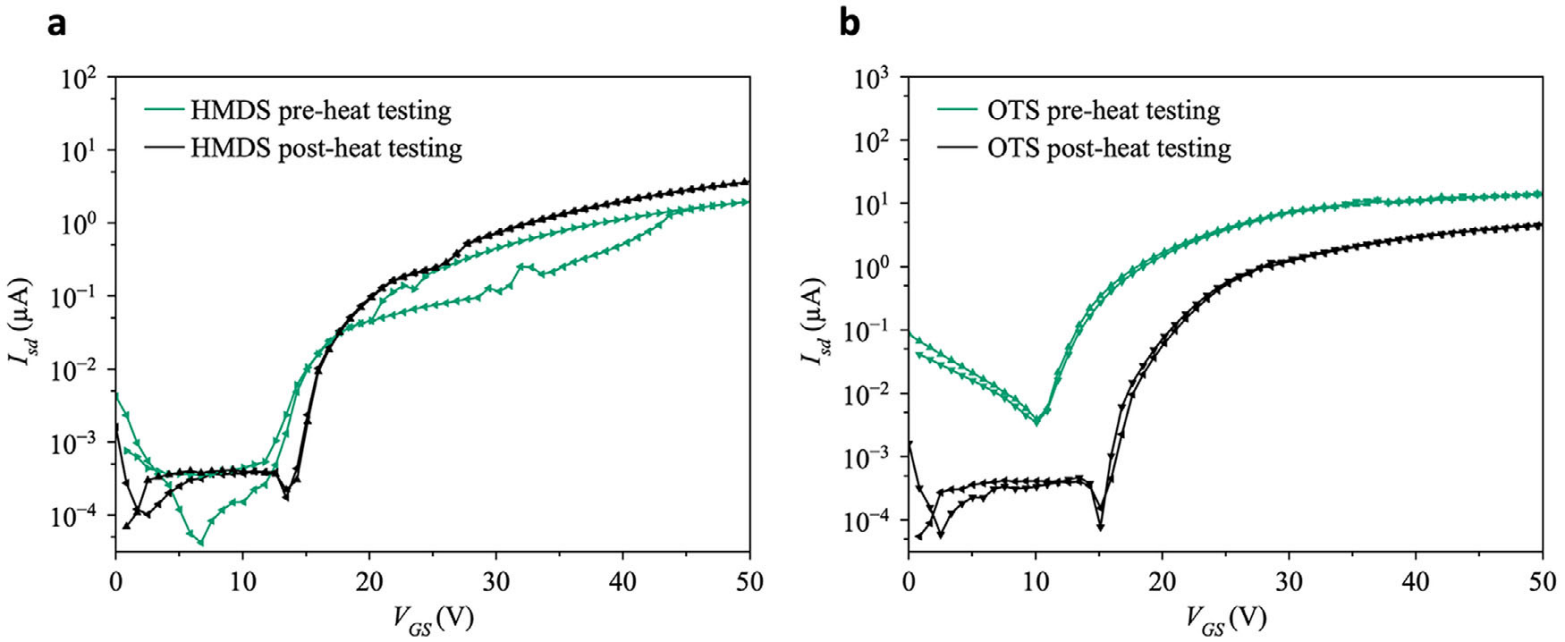
Characteristic transfer curves of F_10_‐SiPc OTFTs before (green) and after (black) heated testing (V_DS_ = 50), deposited on OTS (a), and HMDS (b) treated SiO_2_ at substrate temperatures of 25°C.

To assess thermal operational stability, we compared the percent change in *µ_e_* and *V_th_* for devices tested before heated operation and after devices had cooled back to room temperature (Table 1), providing direct insight into how surface treatment and deposition temperature influence device performance pre‐ and post‐stress. OTS‐treated devices showed a reduction in *µ_e_*, reflecting the thermal rigidity of their highly crystalline films. In contrast, PT‐ and HMDS‐treated surfaces exhibited dramatic enhancements, with *µ_e_* increasing by 100%–800%, consistent with molecular rearrangements toward more favorable crystal packing that enhance charge transport [41, 42]. *V_th_* decreased for plasma‐ and HMDS‐treated devices deposited at 100°C, but increased under all other conditions, underscoring the impact that the substrate temperature during deposition can have on the initial electrical properties, and its evolution under thermal stress (Table 1). Figure 5 shows characteristic transfer curves displaying drain current for OTFTs with OTS and HMDS treatments characterized at room temperature, before and after the heated operation. For crystalline films grown on OTS monolayers, heated testing led to reduced *I_on_* and *µ_e_* and increased *V_th_*, indicating limited ability to recover their initial state. In contrast, more amorphous films on HMDS showed slight performance gains after testing, consistent with recent reports [43, 44].

**TABLE 1:** Pre‐ and post‐heated testing device performance characterization measured at room temperature, for F_10_‐SiPc films deposited on PT, HMDS‐ or OTS‐treated substrates at 25°C and 100°C.

Surface Treatment	Deposition T [°C]	Time relative to heated testing	*V_th_* ^a^ [V]	*µ_e_* ^a^ [10^−2^ cm^2^ V^−1^ s^−1^]	Change in *V_th_* (%)	Change in *µ_e_* (%)	Hysterisis [10^−1 ^V]	N_int_ [cm^−2^]	N#^a^
Plasma	25	Pre	25.0 ± 1.5	0.05 ± 0.01			11	4.0 × 10^11^	12
		Post	27.8 ± 7.0	0.10 ± 0.06	11.2	100^*^	6.8	2.2 × 10^11^	9
	100	Pre	34.1 ± 3.6	0.03 ± 0.01			22	6.1 × 10^11^	11
		Post	8.9 ± 1.4	0.28 ± 0.07	−73.9^*^	833^*^	1.5	4.2 × 10^11^	10
HMDS	25	Pre	11.5 ± 0.9	0.44 ± 0.2			6.4	3.5 × 10^11^	12
		Post	17.6 ± 5.8	1.68 ± 0.4	53.0^*^	281^*^	1.4	2.2 × 10^11^	9
	100	Pre	34.1 ± 3.6	0.04 ± 0.01			4.2	8.0 × 10^11^	12
		Post	15.4 ± 3.4	0.18 ± 0.07	−49.2^*^	350^*^	2.6	1.1 × 10^11^	11
OTS	25	Pre	11.9 ± 1.2	8.52 ± 1.3			3.1	4.4 × 10^11^	10
		Post	17.3 ± 3.6	2.47 ± 2.1	45.4^*^	−71.0^*^	10	2.1 × 10^11^	11
	100	Pre	18.6 ± 1.0	0.52 ± 0.1			3.2	3.4 × 10^11^	9
		Post	15.8 ± 1.5	0.35 ± 0.1	17.7	−32.7	5.5	3.0 × 10^11^	8

^a^ *µ_e_* and *V_th_* were calculated using an average value, measured from N# functional devices. Statistical significance between pre‐ and post‐heating conditions was determined using single‐factor ANOVA (α = 0.05). Significant differences (*p* < 0.05) are indicated by asterisks.

The interfacial trap density (*N*_int_) and hysteresis were extracted from the transfer characteristics. Following a heated operation, a general reduction in *N*_int_ was observed across all devices. While this trend is consistent with thermally activated detrapping, the accompanying evolution of hysteresis and threshold voltage indicates additional interface‐dependent contributions. Consistently, hysteresis decreased after heating for devices with all surface treatments except OTS, suggesting that *V*_th_evolution cannot be attributed solely to trap depopulation. For devices with F_10_‐SiPc films deposited on OTS‐treated SiO_2_ at substrate temperatures of 25°C and 100°C, hysteresis increased from 0.31 to 1.0 V and from 0.32 to 0.55 V, respectively. Similar behavior has been reported for surface treatments with large dipole moments, where thermal exposure induces irreversible changes in hysteresis and threshold voltage, arising from interfacial polarization and dipole‐induced shifts in dielectric–semiconductor energetic alignment, particularly for ODTS‐treated interfaces [45, 46]. By comparison, HMDS‐treated substrates show negligible changes in hysteresis after thermal stress, indicating greater interfacial electrostatic stability. Representative transfer curves before and after heated operation are presented in Figure 5.

### 2.3 Morphology Evolution Grazing‐Incidence Wide‐Angle X‐Ray Scattering (GIWAXS)

**FIGURE 6:**
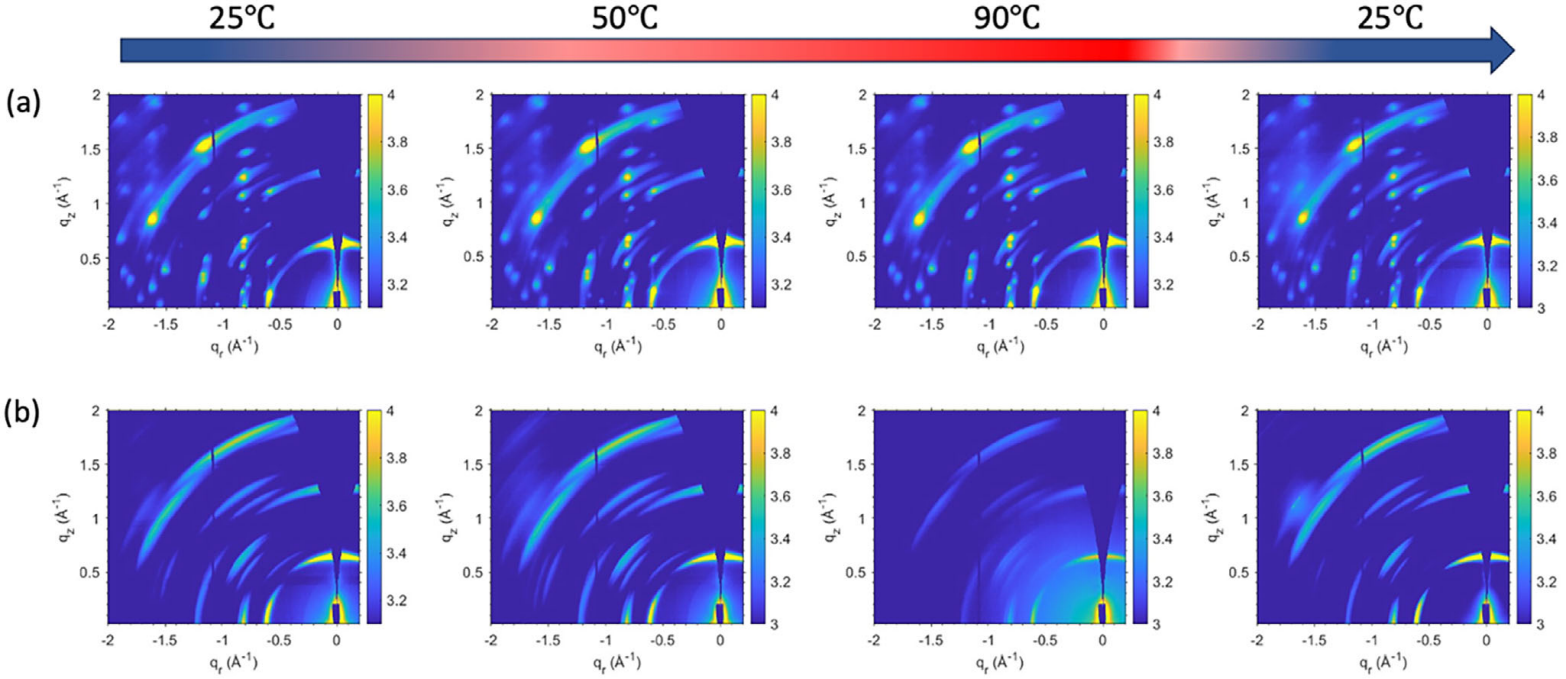
GIWAXS maps recorded at 25°C, 50°C, 90°C, and after thermal cycling cooling for F_10_‐SiPc films deposited by PVD at room temperature on (a) OTS‐treated Si substrates, and (b) HMDS‐treated Si substrates.

Figure 6 shows GIWAXS maps of F_10_‐SiPc films deposited at 25°C on HMDS‐ and OTS‐treated Si surfaces (to avoid SiO_2_ background). OTS‐treated films exhibit numerous sharp spots with minimal χ broadening, indicating good crystalline texture uniformity (Figure 6A), whereas HMDS‐treated films display fewer reflections with highly broadened peaks, consistent with a lower degree of crystallinity and texture uniformity (Figure 6B). Azimuthal integration (*q* cut, Figure S7) of GIWAXS data for F_10_‐SiPc films on OTS‐ and HMDS‐treated substrates matched the powder diffraction pattern of a known polymorph (CCDC DAJMIO). SC‐XRD further revealed that in OTS‐treated films, the strongest high‐q reflections, (222) and (132) at χ = 62°/37.5°, indicate a single preferred orientation with the (010) plane parallel to the substrate and the Pc ring tilted ∼35° toward the substrate. Table 2 demonstrates the difference in morphology and crystalline order between F_10_‐SiPc films deposited on substrates at 25°C and 100°C by quantifying the overall film RMS roughness and coherence length (*L_C_*) values for both the phthalocyanine and axial fluorinated phenyl groups of the F_10_‐SiPc molecule. For films deposited on both HMDS and OTS‐treated substrates, increasing the substrate temperature from 25°C to 100°C during deposition resulted in decreased coherence lengths. While the films deposited at 100°C have larger grains as shown in the AFM, the reduction in *L_C_* indicates a lower degree of long‐range order within the crystallites, due to the crystals having fewer coherently repeating unit cells [47].

**TABLE 2:** RMS^a^ roughness and coherence lengths for F_10_‐SiPc films deposited on HMDS‐ and OTS‐treated Si substrates at room temperature and 100°C.

Surface treatment	Deposition T [°C]	RMS roughness [nm]	Phthalocyanine ring *L_c_* [nm]	Axial fluorinated phenyl ring *L_c_* [nm]
HMDS	25	2.66	9.08	9.71
	100	13.9	5.77	7.40
OTS	25	3.64	9.43	9.44
	100	10.3	8.16	8.57

^a^ Root mean square (RMS) roughness was measured using the AFM apparatus.

To compare film crystalline textures, partial pole figures of the (132) reflection (Figure S5) were analyzed for peak broadening, confirming higher crystallite order on OTS‐treated substrates. Both films display the same preferred orientation but differ in the uniformity of their crystalline texture, with OTS‐treated thin films showing the most uniform one. GIWAXS data for all surface treatments at 100°C (Figure S8) show the same trends.

GIWAXS measurements were conducted in situ at 25°C, 50°C, 90°C, and then cooled back down to 25°C (Figure 6). The OTS‐treated film shows no significant change in peak position, intensity, or broadening with temperature. In contrast, the HMDS‐treated film exhibits marked changes: reflection spot intensity decreases with heating but nearly recovers after cooling to 25°C. Thus, while elevated temperature reduces the degree of crystallinity or crystal coherence in poor, uniformly textured films, the effect is reversible, whereas uniformly textured F_10_‐SiPc films remain unaffected. Surface treatments producing monolayers with higher contact angles, such as OTS [48], are known to promote the formation of more crystalline F_10_‐SiPc films [3]. However, it has been found that in highly ordered films, increasing the temperature leads to a decline in *µ_e_*, consistent with ‘band‐like’ temperature dependence of mobility [43, 49, 50, 51]. This behavior can also arise from increased interfacial charge trapping due to the strongly dipolar OTS monolayer, which limits charge carrier transport and contributes to the non‐recoverable mobility decline after heating [46]. Conversely, surface treatments that result in lower contact angles, such as HMDS or bare SiO_2_ treated with plasma, produce more amorphous semiconducting films [52, 53]. Amorphous films show a differing temperature dependence from crystalline films, with *µ_e_* slightly increasing as temperature rises, revealing that thermally activated behavior dominates charge transport [44]. As temperature increases, the additional thermal energy enables carriers to overcome energetic barriers more easily between these states, enhancing the efficiency of the charge hopping process and leading to modest improvements in *µ_e_* [47]. The increase in conductivity observed for the HMDS films can be described by the multiple trapping and release (MTR) model [54], in which charge transport follows an Arrhenius‐type temperature dependence, $\mu_{e}\approx e^{\frac{E_{a}}{K_{B}T}}.$ The activation energy (*E_a_*) obtained from the slope of the logarithmic plot (Figure S9) was 271 meV, which is in good agreement with values previously reported for organic semiconductors exhibiting thermally activated charge hopping transport [55, 56]. Overall, this surface‐treatment‐dependent capacity for thermally induced structural change underlines the variations in electrical behavior at different operating temperatures and demonstrates the importance of semiconductor film morphology and choice of interlayer surface treatment when designing for thermal operational stability of an OTFT.

## 3 Conclusion

This study highlights the tunable nature of F_10_‐SiPc OTFTs through careful control of interfacial surface treatment and deposition temperature, offering a versatile approach to optimize device performance for high‐temperature applications. OTS‐treated substrates produce highly crystalline films with excellent room‐temperature mobility but reduced performance at elevated temperatures, whereas HMDS‐ and plasma‐treated substrates yield less crystalline films with worse initial electrical performance but show an increase in performance that was maintained after operating at high temperatures. The decoupling of deposition temperature and surface treatment effect on thermal stability is studied by in situ electrical characterization of a rich amount of data (more than 10 devices per condition). The results show that *V_th_* evolution at high temperature is more dependent on initial substrate fabrication temperature. In contrast, interfacial surface treatments strongly effects mobility and hysteresis changes, likely arising from the strong dipole moment of OTS in comparison with HMDS and PT treatments. In situ GIWAXS and AFM analyses reveal that these fabrication parameters dictate the degree of crystallinity, structural rearrangement, and thermal response of the films. Collectively, these results demonstrate that OTFT performance can be strategically tuned through interlayer selection and deposition conditions, providing a scalable framework for designing thermally robust organic electronics tailored to specific operational requirements.

## 4 Methods

### 4.1 Materials

All solvents used for synthesis and device fabrication were ACS grade and were used without any further purification. All silanes were purchased from Sigma–Aldrich and used as received. F_10_‐SiPc was synthesized using previously reported protocols [57].

### 4.2 Device Fabrication

N‐doped silicon substrates with 230 nm of thermally grown oxide were first washed sequentially with acetone and isopropanol and dried with nitrogen. The substrates were then sequentially sonicated in detergent, DI water, acetone, and methanol for 5 min each, and dried with nitrogen. The substrates were then oxygen plasma‐treated for 15 min under vacuum to clean and hydrolyze the surfaces. Substrates to be treated with OTS were then washed with water and isopropanol, before being treated in a solution of 1% v/v OTS in toluene at 70°C for 1 h. OTS‐treated substrates were then removed from the solution, washed with toluene, and dried with nitrogen before being thermally annealed at 70°C for 1 h under vacuum. Substrates to be treated with HMDS were transferred to a wet nitrogen glove box after plasma treatment. 50 µL of HMDS was then statically spin‐coated on the substrates at 3000 rpm for 30 s. These substrates were then thermally annealed at 150°C for 1 h under vacuum. Plasma, OTS, and HMDS treated substrates were transferred into a dry nitrogen glovebox connected to a PVD chamber with a nominal vacuum level of 10^−6^ Torr, where a 50 nm‐thick layer of F_10_‐SiPc was thermally deposited at a rate of 0.2 Å s^−1^. Before the deposition of the electrodes, two corners along the width of the substrate were scratched with a diamond‐tipped pen to expose the Si base, allowing the gate electrode deposition. The substrates were then placed in shadow masks with a channel length (L) of 30 µm and a channel width (W) of 1000 µm, with 20 individual transistors per substrate. The source‐drain electrodes consisting of a 10 nm manganese (0.5 Å s^−1^) interlayer and 50 nm of silver (2 Å s^−1^) were deposited by PVD.

### 4.3 Device Characterization

OTFTs were transferred to and tested in a separate nitrogen‐filled glovebox with oxygen and moisture levels below 3 ppm. Electrical characterization was carried out using a custom‐built Gen 1H auto tester and a Keithley 2614B source meter controlled with a custom LabVIEW software for setting and sweeping voltages. Output curves were plotted by measuring the drain current (*I_SD_*) while sweeping the source drain voltage (*V_SD_*) from 0 to 50 V and maintaining the gate voltage (*V_GS_*) at a certain voltage between 0 and 50 V, stepping in intervals of 10 V. Transfer curves were generated by measuring the I_DS_ while maintaining a constant *V_DS_* and sweeping the *V_GS_* both forward and backward from 0 to 50 V. The sweeping of transfer curves was conducted four times to ensure device stability. *V_th_* and *µ_e_* values are calculated from the final three transfer curves and averaged for each device [27]. This study employs a custom Python code developed by a member of the Lessard Research Group to calculate accurate *V_th_* and *µ_e_* values. The code enables the user to rapidly sort through the transfer curve plots and apply the MOSFET model to fit the data and acquire accurate *V_th_*, m, and *I*_ON/OFF_ values for each device. The code takes the data from the auto tester and plots each transfer and output curve, including the $\sqrt{I_{\mathrm{SD}}}$ curve. The code will also determine whether to test in the linear or saturation region, such that the linear region equations will be applied if V_SD _ < (V_GS_ − V_th_) and the saturation region equations will be applied if V_SD _ > (V_GS_ − V_th_). Once the code has plotted transfer and output curves, the user will modify the V_GS_ range to determine an accurate *V_th_* value and correct the slope of the $\sqrt{I_{\mathrm{SD}}}$ curve used to calculate the *µ_e_* value with the MOSFET model. Sample transfer and output curves are demonstrated in Figure S4. The following equations are used to calculate the device's performance characteristics:

If the test is being done in the linear regime, Equation (1), shown below, will be used to calculate the mobility:

(1) $$\mu_{e}=\frac{LV_{SD}}{WC_{i}}\frac{\sqrt{I_{SD}}}{V_{SG}}$$

If the test is being done in the saturation regime, which is the case in this study, Equation (2) will be used to calculate the mobility:

(2) $$\mu_{e}=\frac{2L}{WC_{i}}{\left(\frac{\sqrt{I_{SD}}}{V_{SG}}\right)}^{2}$$

Where *L* and *W* represent the length and width of the device channel, respectively. *C_i_* represents the capacitance of the dielectric, which is calculated with Equation (3) for each regime:

(3) $$C_{i}=ke_{0}\left(\frac{A}{d}\right)$$

Where k is the dielectric constant of the material, *e*_0_ is the vacuum permittivity, and A is the surface area of the dielectric.

### 4.4 AFM

AFM images were taken using a Bruker Dimension FastScan AFM with ScanAsyst‐Air tips in PeakForce Tapping Mode. Imaging processing was performed with NanoScope Analysis v.3.0. The samples per line used were 512.

### 4.5 GIWAXS

Grazing incidence wide‐angle X‐ray scattering (GIWAXS) data were collected at Synchrotron SOLEIL on the SIRIUS beamline. A beamline energy of ∼10 keV (wavelength = 1.24 Å) was selected, and all images were collected at a grazing incidence angle of 0.2°. The sample position was oriented using an automated, iterative procedure to scan the sample height and incidence (theta) angle. WAXS data were collected on a Pilatus detector using a detector distance of ∼312 mm to obtain a *Q* value of 2.2. The sample‐detector distance and tilt were calibrated using a silver behenate reference material and confirmed with a poly(3‐hexyl thiophene) (P3HT, Rieke Metals) standard. Samples and reference materials were collected with an acquisition time of 10 s, and 11 images were collected and averaged. All data were analyzed using the GIXSGUI software package in MATLAB [58]. To remove the horizontal “dead zones”, two images with a vertical offset of 23 pixels were obtained for each sample, and the “Gap Fill” function was utilized in GIXSGUI.

Coherence length values were calculated using the Scherrer relation [47], shown in Equation (4):

(4) $$L_{C}=\frac{2\pi K}{\Delta q}$$

Where *L_C_* is the coherence length (nm), *K* is the shape factor taken to be 0.9 [59], and Δ*q* is the full width at half‐maximum of an azimuthal q‐cut diffraction peak (nm^−1^).

### 4.6 Statistical Analysis

All reported OTFT performance characterization values represent the average of 9–12 devices (*n* = 9–12). Only non‐functioning devices were removed, without any outlier removal. Results were presented as mean ± standard deviation (SD). Statistical analyses comparing pre‐ and post‐heated testing were performed using one‐way ANOVA, implemented in Microsoft Excel. Statistical significance was set at α = 0.05.

## Conflicts of Interest

The authors declare no conflicts of interest.

## Supporting information




**Supporting File**: smtd70571‐sup‐0001‐SuppMat.docx.

## Data Availability

The data that support the findings of this study are available from the corresponding author upon reasonable request.
