# Supplementary material for: Thermal Operational Stability in Organic Thin Film Transistors: The Critical Role of Interface Composition and Deposition Conditions
Source: Small Methods. 2026 Feb 28;10(12):e02171. doi: 10.1002/smtd.202502171 (PMC13288016; doi:10.1002/smtd.202502171)
Supplement: Supplementary file 1 — Supporting File: smtd70571‐sup‐0001‐SuppMat.docx. [file SMTD-10-e02171-s001.docx]

Thermal Operational Stability in Organic Thin Film Transistors: The Critical Role of Interface Composition and Deposition Conditions

Forest St-Denis Weintrager^1,¥^, Sofia Gallardo-Pascual^1,¥^, Nicolas Ledos^1,2^, Raluchukwu B. Ewenike^1^, Audithya Nyayachavadi^1^, Halynne R. Lamontagne^1,2^, Benoît H. Lessard^1,3*^

*^1^Department of Chemical and Biological Engineering, University of Ottawa, 161 Louis Pasteur, Ottawa, ON, Canada, K1N 6N5*

*^2^Department of Chemistry and Biomolecular Sciences, University of Ottawa, 150 Louis Pasteur, Ottawa, ON, Canada, K1N 6N5*

*^3^School of Electrical Engineering and Computer Science, University of Ottawa, 800 King Edward Ave, Ottawa, ON, Canada, K1N 6N5*

^¥^ These authors contributed equally.

*Corresponding Authors: [Benoit.Lessard@uottawa.ca](mailto:Benoit.Lessard@uottawa.ca) (BHL)

***Supporting Information***


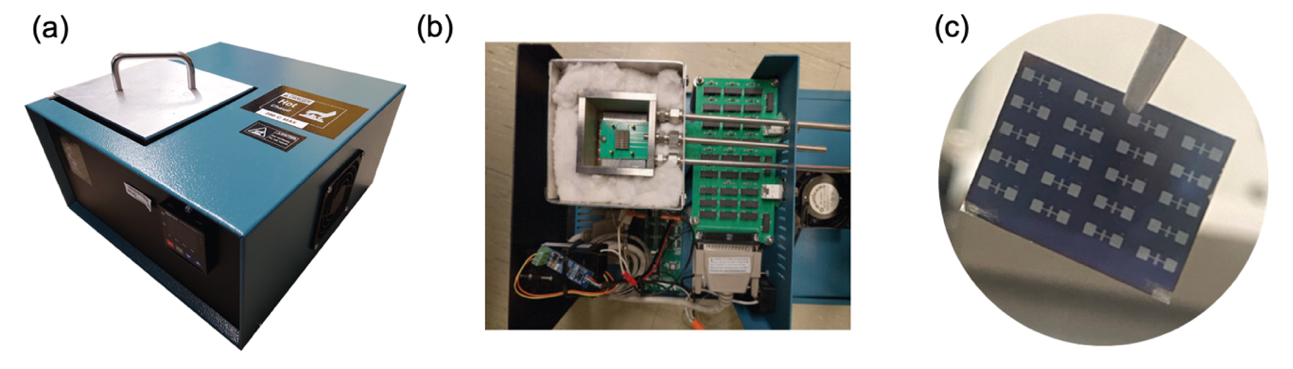


**Figure S1.** a) Heated auto-tester, b) Heated chamber and electronic connections c) Substrate holding 20 OTFTs.

**
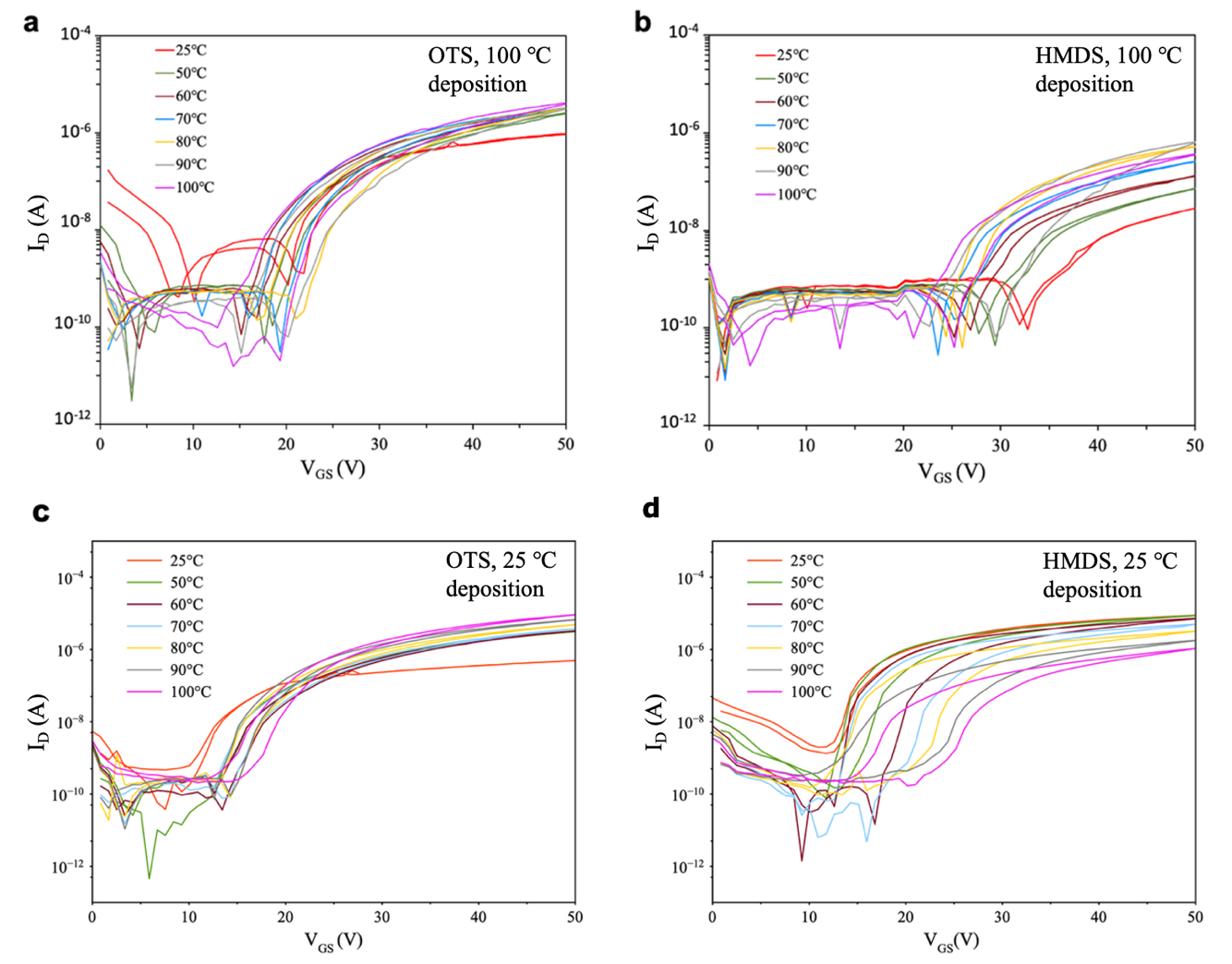
**

**Figure S2.** Characteristic transfer curves measured at operating temperatures from 25 ℃ to 100℃ for OTFTs with F_10_-SiPc deposited on a) OTS and b) HMDS treated substrates at 100 ℃, c) OTS and d) HMDS treated substrates at 25 ℃.


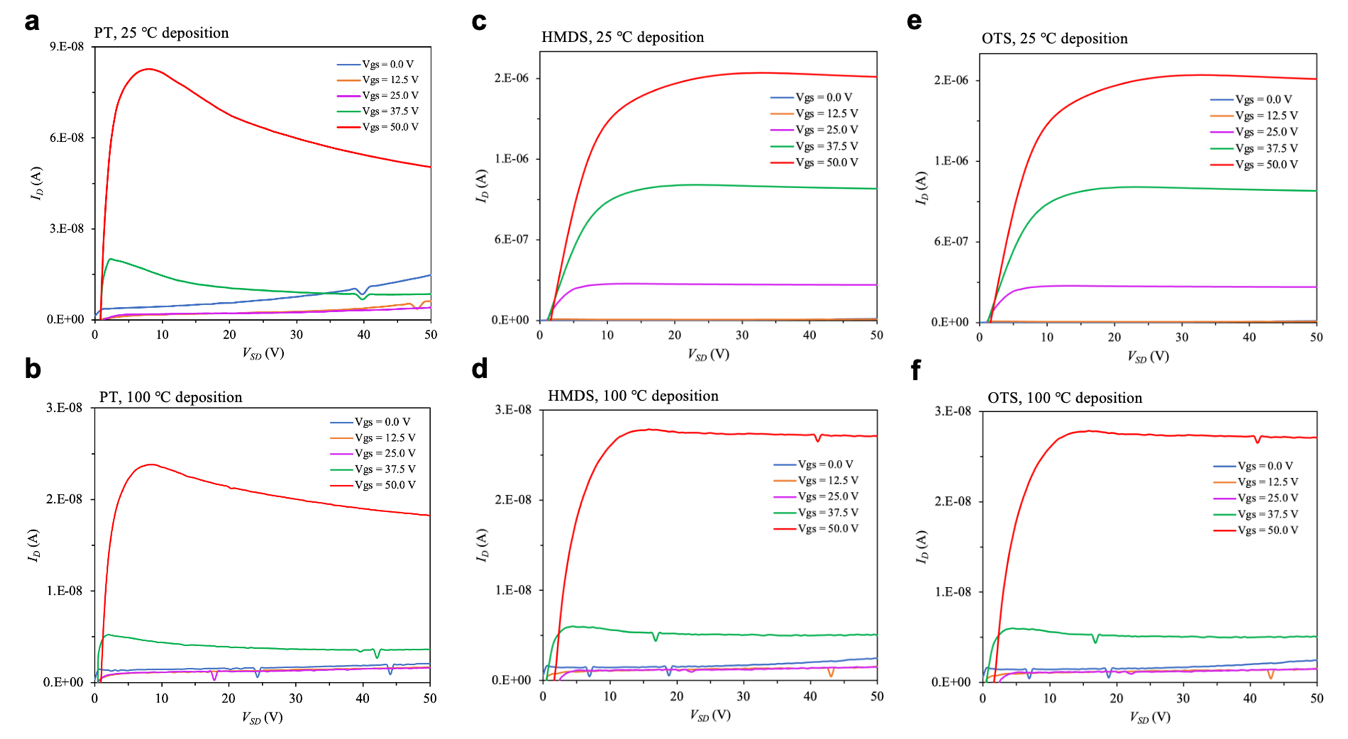


**Figure S3.** Characteristic output curves measured at room temperature for OTFTs with F_10_-SiPc deposited on SiO_2_ substrates with treatments of (a,b) plasma at substrate temperatures of 25 ℃ and 100℃ during deposition, (c,d) HMDS at substrate temperatures of 25 ℃ and 100℃ during deposition, and (e,f) OTS at substrate temperatures of 25 ℃ and 100℃ during deposition.

**
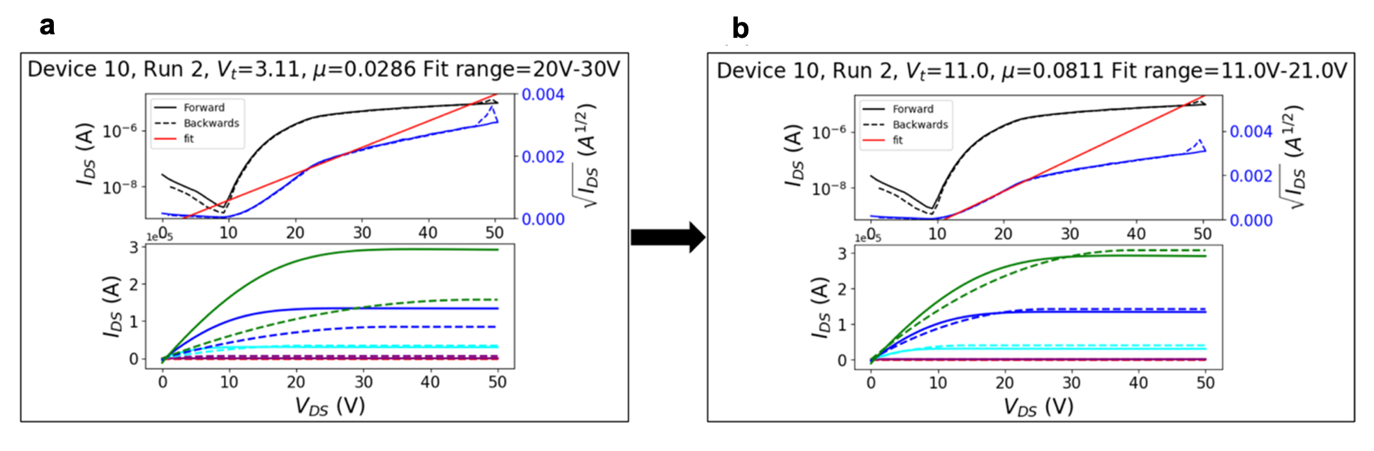
**

**Figure S4**. MOSFET model fitting for code methodology for a) original plot, b) fitted plot.

**
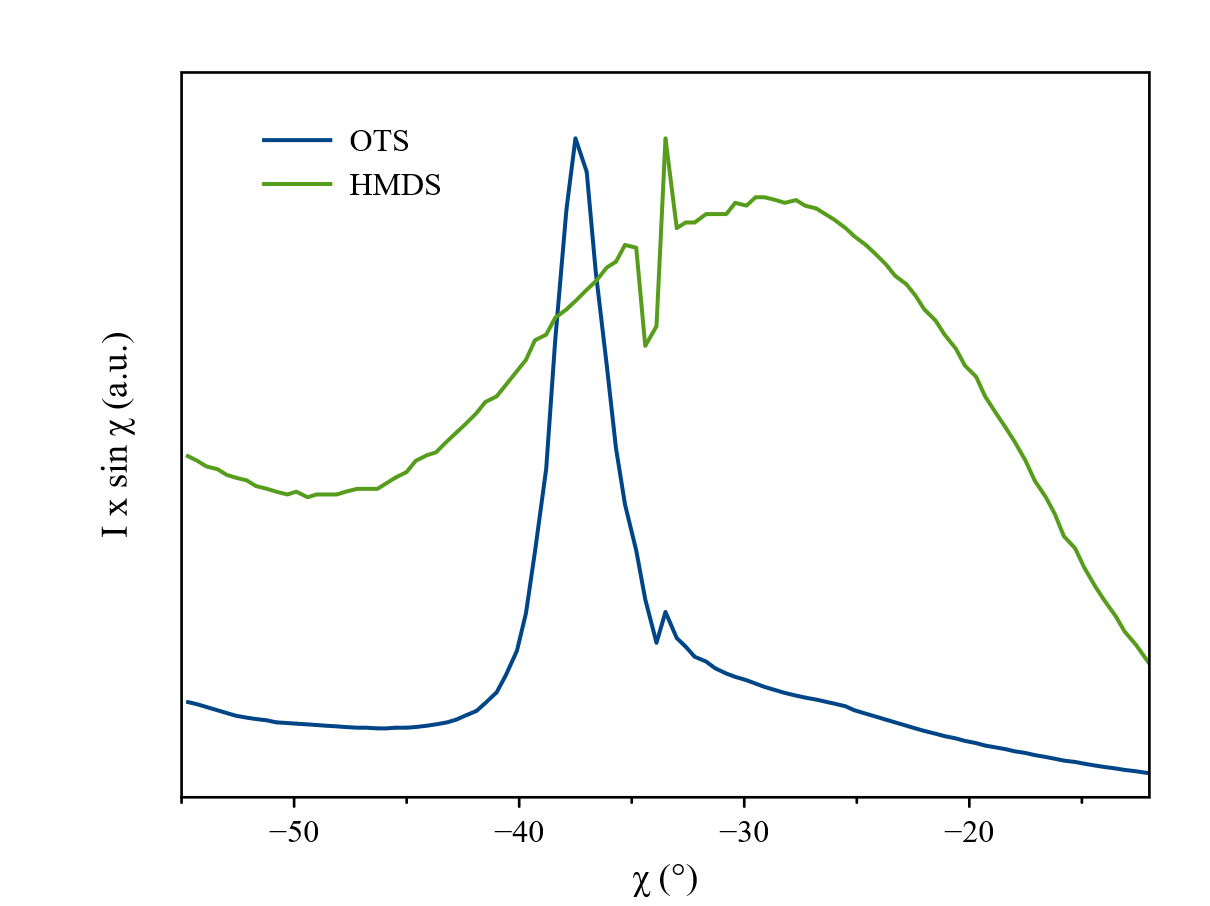
**

**Figure S5**. Normalized GIWAXS linear *χ* cuts for F_10_-SiPc films deposited on OTS and HMDS treated Si substrates, measured at room temperature.


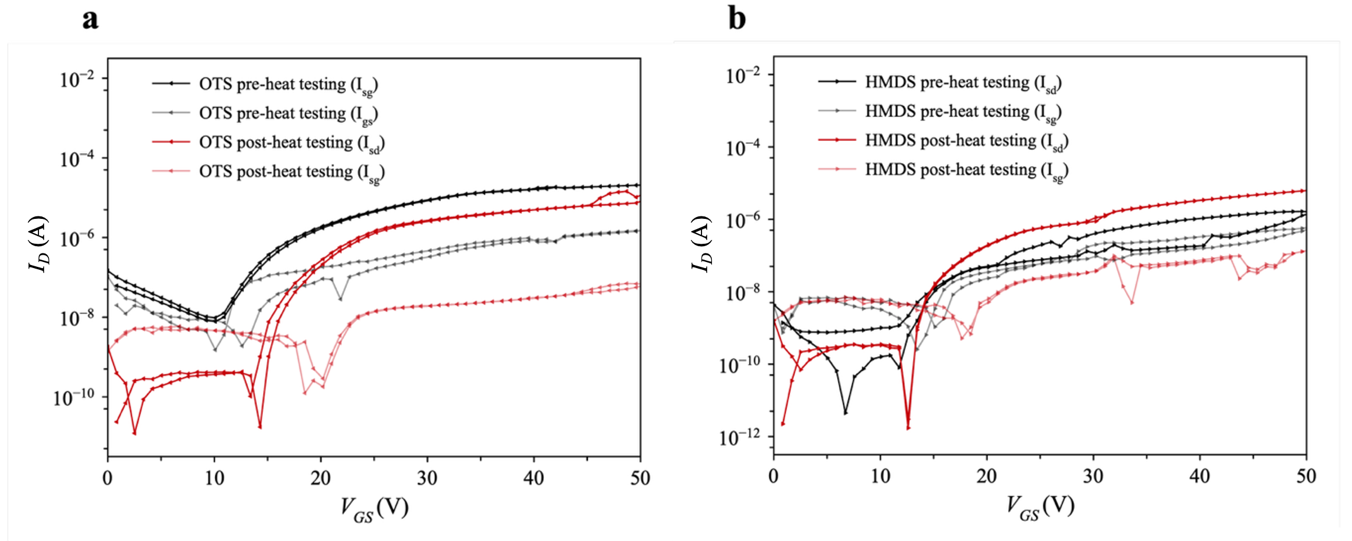


**Figure S6.** Characteristic transfer curves for F_10_SiPc OTFTs displaying drain current before heated-testing and after cooling back to room temperature for devices with F10-SiPc deposited on (a) OTS and (b) HMDS treated films.


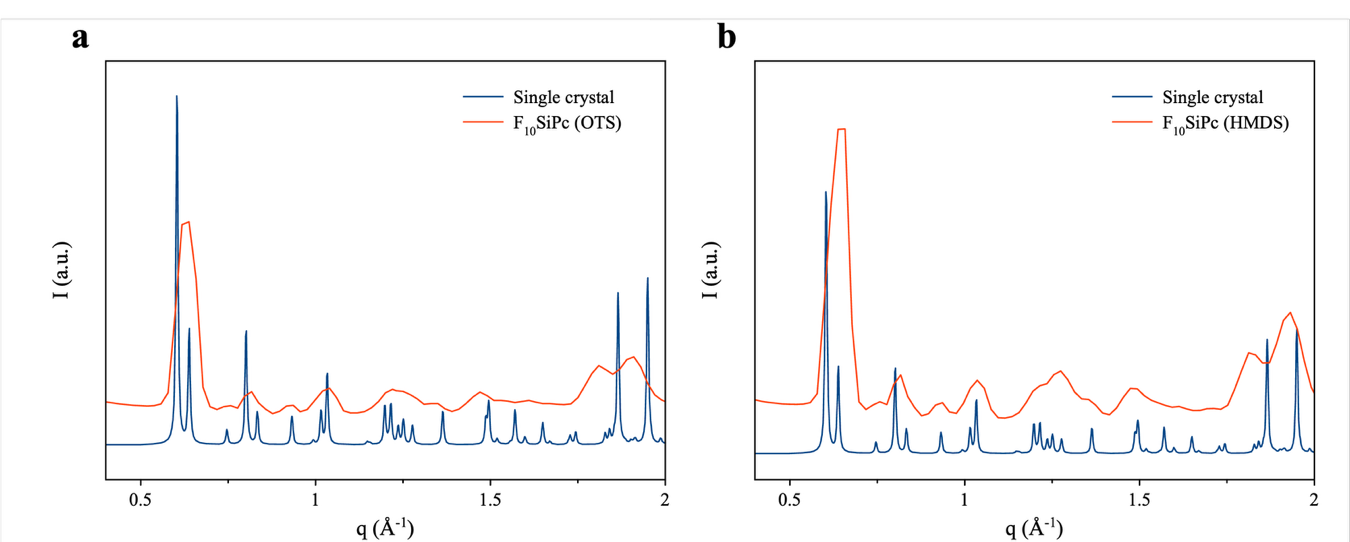


**Figure S7.** GIWAXS azimuth linecuts of F10-SiPc deposited on SiO_2_ with surface treatments a) OTS and b) HMDS against single crystal X-ray diffraction pattern of F10-SiPc (CCDC #1034275).


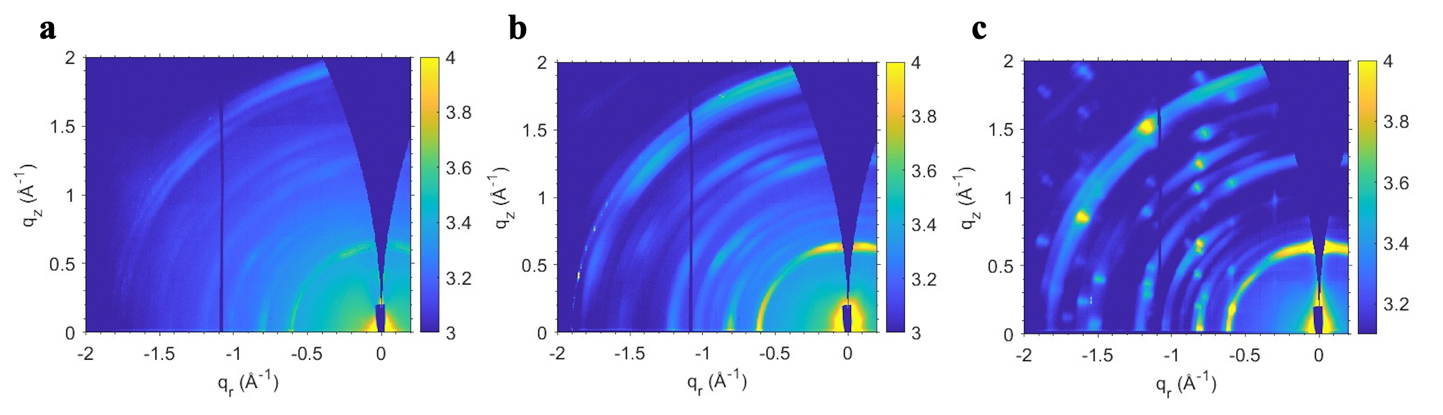
**Figure S8.** GIWAXS spectra recorded at a temperature of 100 ℃ for F_10_-SiPc films deposited on a) plasma, b) HMDS, and c) OTS treated Si substrates.


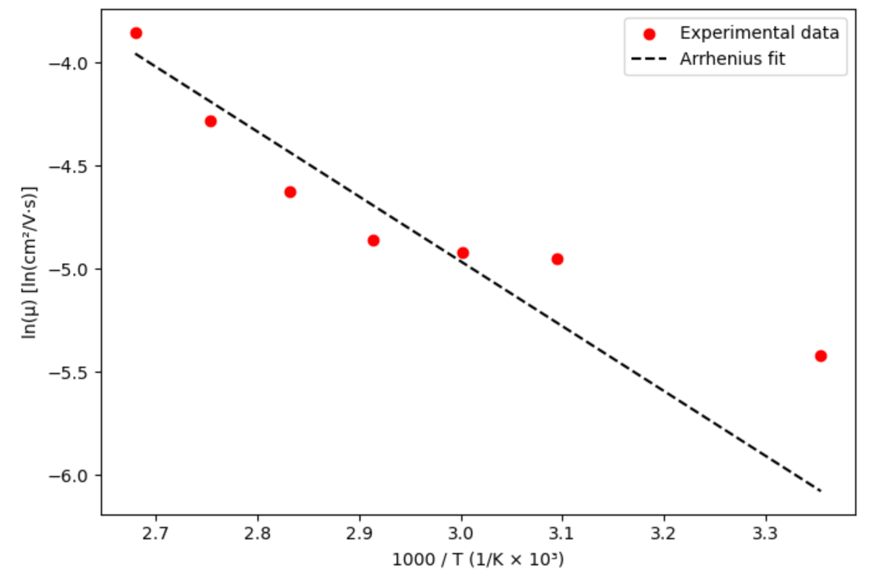


**Figure S9.** Arrhenius plot of the field-effect mobility (μ) for HMDS-treated OTFTs. The natural logarithm of the mobility is plotted as a function of the inverse temperature (1000/T).
